# Assessment of Two Different Glucagon Assays in Healthy Individuals and Type 1 and Type 2 Diabetes Patients

**DOI:** 10.3390/biom12030466

**Published:** 2022-03-18

**Authors:** Martina Brunner, Othmar Moser, Reingard Raml, Maximilian Haberlander, Beate Boulgaropoulos, Barbara Obermayer-Pietsch, Eva Svehlikova, Thomas R. Pieber, Harald Sourij

**Affiliations:** 1 CF-Clinical Trials Unit, Center for Medical Research, Medical University of Graz, 8036 Graz, Austria; martina.brunner@medunigraz.at; 2 Division of Endocrinology and Diabetology, Department of Internal Medicine, Medical University of Graz, 8036 Graz, Austria; othmar.moser@medunigraz.at (O.M.); maxihaberlander@googlemail.com (M.H.); beate.boulgaropoulos@medunigraz.at (B.B.); barbara.obermayer-pietsch@medunigraz.at (B.O.-P.); eva.svehlikova@medunigraz.at (E.S.); thomas.pieber@medunigraz.at (T.R.P.); 3 Division Exercise Physiology and Metabolism, Department of Sport Science, University of Bayreuth, 95440 Bayreuth, Germany; 4 Joanneum Research Forschungsgesellschaft mbH HEALTH—Institute for Biomedicine and Health Sciences, 8010 Graz, Austria; reingard.raml@joanneum.at; 5 Interdisciplinary Metabolic Medicine Trials Unit, Medical University of Graz, 8036 Graz, Austria

**Keywords:** glucagon assay, glucagon, ELISA, RIA, type 1 diabetes mellitus, type 2 diabetes mellitus

## Abstract

Methods for glucagon analysis suffered in the past from lack of specificity and a narrow sensitivity range, which has led to inaccurate results and to the suggestion that type 1 diabetes (T1D) and type 2 diabetes (T2D) patients have elevated fasting glucagon levels. However, the availability of more specific and more sensitive methods to detect intact glucagon has shown that actual glucagon levels are lower than previously assumed. This study aimed to characterize fasting plasma glucagon levels in healthy individuals and T1D and T2D patients with two different glucagon assays. The study included 20 healthy individuals, 20 T1D and 20 T2D patients. Blood was collected under fasting conditions. A double-antibody sandwich enzyme-linked immunosorbent assay (ELISA) and a conventional radioimmunoassay (RIA) were used. A significant difference in fasting glucagon levels between healthy individuals and T2D was observed by ELISA, but not by RIA. ELISA also yielded lower glucagon levels in healthy individuals than in T1D and T2D patients which RIA did not. RIA produced significantly (*p* = 0.0001) higher overall median glucagon values than ELISA in a pooled analysis. These results underline the notion that the choice of selective laboratory methods is highly relevant for mechanistic endocrine research.

## 1. Introduction

The accurate measurement of glucagon is highly relevant in endocrine research [1,2,3] as accurate interpretation of data depends on reliable glucagon measurements. Dysfunction of alpha- and beta-cells in type 1 diabetes (T1D) and type 2 diabetes (T2D) patients manifests as inappropriate glucagon response [1,2], and during hypoglycaemia glucagon has been identified as a major player during counter-regulation [4]. Based on findings from different methods for glucagon analysis, elevated fasting glucagon levels have been suggested both in T1D [5,6,7] and T2D patients [6,8,9,10,11].

The significance of glucagon level data, however, is determined by sufficient specificity, as well as by an adequate sensitivity range of the used assay [12].

It is a special challenge to specifically detect intact glucagon without also measuring glucagon-related molecules with the same amino acid sequence as glucagon. Glucagon is predominantly produced in the pancreatic alpha-cells [13], deriving from the precursor molecule proglucagon that is expressed in the pancreatic alpha-cells, in the intestine and the brain. In the pancreatic alpha-cells the enzyme prohormone convertase 2 mediates the formation of glucagon, glicentin-related polypeptide, an intervening peptide (IP-1) and the major proglucagon fragment. In the intestine and the brain, the enzyme prohormone convertase 1/3 mediates the formation of glucagon-like peptide-1 (GLP-1), glucagon-like peptide-2 (GLP-2), an intervening peptide (IP-2), and glicentin, which can further be cleaved into glicentin-related polypeptide and oxyntomodulin [13,14,15,16,17,18,19]. Of note, glicentin and oxyntomodulin comprise the entire amino acid sequence of intact glucagon [16,17]. Particularly, the potential cross-reactivity with these glucagon-related molecules with the same amino acid sequence as intact glucagon poses major challenges for the accurate measurement of plasma glucagon levels, as the commonly used mostly unspecific assays may also respond to such cleavage products [17,18,19,20], and may, as a consequence, produce falsely high glucagon concentrations. A high specificity is mainly achieved by using two antibodies in a sandwich-approach instead of only one [21].

Apart from the specificity of the assay used, the sensitivity, especially in the lower glucagon concentration regions, is an important factor that contributes to reliable data [12]. The existing already low glucagon concentrations in blood are further decreasing in response to increasing glucose levels (e.g., after meal intake), and the ability of the assays to register this decline in glucagon concentrations based on already low values is of importance for the resulting glucose tolerance in patients. Reliable measurement of glucagon concentrations in this concentration range is, thus, of high relevance in endocrine research [20]. Another aspect that contributes to challenges in accurate glucagon measurement is the fact that immunoreactive glucagon is highly susceptible to proteolytic degradation in the collected blood samples [20,22,23,24,25].

The availability of more specific methods and of methods with a higher sensitivity especially in the lower glucagon concentration ranges, such as the highly specific glucagon sandwich ELISA which detects exclusively intact glucagon [21,26], and also the availability of blood collection tubes that are optimized for glucagon stabilization [27,28], have indicated that actual glucagon levels may be lower than previously assumed.

Our study aimed to characterize fasting plasma glucagon levels in healthy individuals and both patients with T1D and T2D using a highly specific and sensitive glucagon assay (dual antibody double sandwich ELISA) and a conventional radioimmunoassay (RIA using only one antibody). In contrast to the conventional radioimmunoassay (RIA) which uses only one antibody, the ELISA uses two specific antibodies raised against the N-terminal and C-terminal region of glucagon [12,26].

## 2. Materials and Methods

We performed a single centre, prospective cohort study to assess fasting glucagon levels in healthy individuals and in type 1 and 2 diabetes patients with two different glucagon assays (ELISA and RIA). The study was approved by the Ethics Committee of the Medical University of Graz, Austria (registration number: 28-233 ex 15/16), registered at the German Clinical Trials Register (DRKS00022061) and conducted in accordance with the Declaration of Helsinki and Good Clinical Practice, designated Standard Operation Procedures and with laws and regulations relevant to clinical trials in Austria [29,30]. All individuals gave oral and written informed consent before any trial-related activities were started.

### 2.1. Study Participants

For the assessment of basal glucagon levels, 60 individuals (34 male and 26 female) were included, comprising 20 healthy individuals, 20 T1D and 20 T2D patients. The diagnostic criteria for T1D and T2D were set according to the American Diabetes Association (ADA) [31]. Both, male and female (age ≥ 18 years) were eligible for this analysis and the three groups were not matched for specified characteristics. Patients with T2D were eligible if they were on either diet or a monotherapy or combination of insulin, sodium-dependent glucose cotransporter-2 (SGLT-2) inhibitors, metformin, dipeptidyl peptidase IV (DPP IV) inhibitors, or sulfonylurea. Patients with T1D were insulin-dependent and treated with either multiple daily injections or continuous subcutaneous insulin infusion.

### 2.2. Experimental Procedures

All study participants donated blood either during the routine visit at the outpatients’ clinic or at the study visit. All participants attended the visits in fasting condition. Venous blood samples were collected using BD P800 blood collection tubes optimized for glucagon stabilization by means of additives, such as of DPP IV, esterase, and protease inhibitors (Becton, Dickinson and Company Vacutainer System, Franklin Lakes, NJ) [27,28]. The samples were centrifuged for 15 min at 1200× *g* and 4 °C immediately after collection, and stored at below –60 °C. The samples were handled and processed as described previously [28]. The two used assays were ELISA (double-antibody sandwich ELISA, cat# 10-1271-01, Mercodia, Uppsala, Sweden) and RIA (Millipore Glucagon RIA MP Biomedicals, Solon, OH, USA). Both assays were stored and carried out according to the manufacturers’ instructions.

### 2.3. Calculations and Statistics

Statistical analyses were conducted using SPSS software v23.0 (SPSS Inc., Chicago, IL, USA) and Graph Pad Prism 8 (GraphPad Software, La Jolla, CA, USA). Data were tested for normal distribution via a Shapiro–Wilk normality test and were reported as mean ± standard deviation (SD) or median (interquartile range). Overall glucagon data and data stratified for healthy individuals, T1D and T2D patients were compared for glucagon assessments by ELISA vs. RIA by means of two-way ANOVA with Sidak’s multiple comparison test. Furthermore, data were analysed using the Bland–Altman method. The percentage of difference between the two glucagon assays was compared between healthy individuals and patients with T1D and T2D by a Kruskal–Wallis test with Dunn‘s multiple comparison test (*p* ≤ 0.05).

## 3. Results

### 3.1. Assessment of Baseline Characteristics

Significant differences were found in comparison of the three groups (Healthy, T1D, T2D) for gender distribution, age, and fasting plasma glucose (*p* < 0.05) (Table 1). There was no significant difference in diabetes duration and HbA1c between T1D and T2D patients.

### 3.2. Assessment of Fasting Glucagon Levels for Healthy Individuals and T1D and T2D Patients

**Figure 1:**
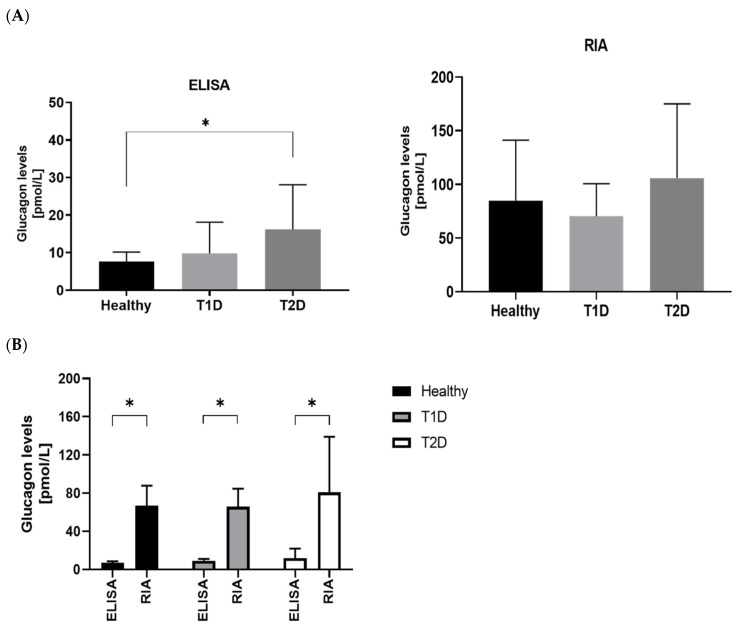
Glucagon levels in healthy individuals, T1D and T2D patients assessed with ELISA and RIA. * *p* = 0.005 (**A**); * *p* < 0.001 (**B**).

Median (IQR) glucagon levels were significantly different between healthy individuals and T2D patients when assessed by ELISA (*p* = 0.005), while according to RIA measurements, no significant differences were found between the groups (*p* = 0.13) (Figure 1A). ELISA yielded lower median glucagon levels in healthy individuals than in T1D and T2D patients, which RIA did not (Figure 1A).

In all groups (Healthy, T1D, T2D) overall median (IQR) glucagon levels were significantly higher when measured with RIA than with the ELISA (72.13 pmol/L [53.28–90.57 pmol/L] vs. 8.55 pmol/L [5.90–12.35 pmol/L], *p* < 0.001). When assessing the mean difference (95% Confidence Interval) between the two glucagon assays, RIA showed significantly higher glucagon levels in healthy individuals [77.12 pmol/L [47.35 to 106.9 pmol/L)], patients with T1D [60.47 pmol/L (30.70 to 90.23 pmol/L)], and T2D patients [89.74 pmol/L (59.97 to 90.23 pmol/L)] (all *p* < 0.0001) than ELISA (Figure 1B).

**Figure 2:**
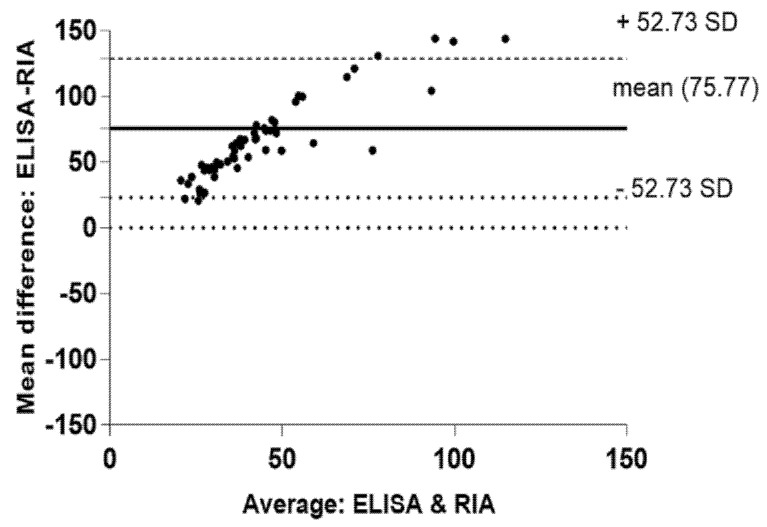
Bland–Altman plot. *x*-axis: The average (pmol/L) of ELISA and RIA for each plasma sample; *y*-axis: The mean difference (pmol/L) of ELISA and RIA. The solid line indicates the mean difference (bias) between the methods. The dashed line and the upper dotted line indicate the limits of agreement between the two methods (95% limits of agreement).

The median glucagon values derived from RIA measurements were significantly higher than those obtained from ELISA in a pooled analysis (Figure 1B), which demonstrated heteroscedasticity in the Bland–Altman plot (Figure 2). The mean positive bias demonstrated by the Bland–Altman analysis was +75.77 pmol/L.

## 4. Discussion

In the present study, we characterized fasting plasma glucagon levels in healthy individuals and patients with T1D and T2D by means of the two glucagon assays ELISA and RIA.

Our results showed that ELISA was able to detect a significant difference in fasting glucagon levels between healthy individuals and T2D, which RIA did not. Furthermore, ELISA yielded lower glucagon levels in healthy individuals than in T1D and T2D patients in contrast to RIA. In a pooled analysis, RIA produced significantly (*p* = 0.0001) higher median glucagon values than ELISA.

Our results are in good agreement with those from previous studies, which have also reported that glucagon levels might be overestimated when measured with RIA compared to ELISA [26,32,33]. Additionally, consistent with previously published data [12,26,34,35], we document that sufficient specificity and an adequate sensitivity range especially in the lower glucagon concentration region are crucial to avoid misinterpretation of data derived from clinical studies [12,26,34,35]. Therefore, the use of such specific and sufficiently sensitive glucagon assays is essential when evaluating dynamic changes in glucagon secretion, e.g., during hypoglycaemia [4,12,36].

The differences in glucagon concentrations obtained from both assays can be at least partially explained by potential cross-reactivity with other glucagon-related molecules [12,21,26,34,35]. We found that RIA-based glucagon levels were in general higher than those obtained from ELISA measurements for each population (Healthy, T1D, T2D).

Our results for glucagon levels obtained with the ELISA for T1D patients are comparable with the results published by Kawamori et al. who re-evaluated plasma glucagon levels in 77 Japanese T1D patients [37].

ELISA was able to detect significant differences in glucagon levels between healthy individuals and T2D patients, which RIA did not. These outcomes are partly in line with the findings from Kobayashi et al. who used ELISA and RIA to assess fasting glucagon levels in healthy individuals and patients with T2D. They found significant differences between both groups when using ELISA, and—in contrast to our results—also when using RIA, although those differences assessed with RIA were less significant [38].

The significant differences in glucagon levels between healthy individuals and T2D patients we found might be explained by the lower standard deviation in the ELISA data and the higher specificity of the ELISA assay which is mainly achieved by the presence of two antibodies raised against the N-terminal and the C-terminal region in a sandwich-like design [12,26,36]. An assay with a C-terminal directed antibody only, which is not exclusively specific for intact glucagon, could also detect truncated molecules, degradation products and glucagon related peptides [24,26,39]. This underlines the fact that the use of specific assays is essential to detect physiological differences and allow accurate interpretation of the resulting data.

We included also T2D patients that were using DPP IV inhibitors and GLP-1 receptor agonists in this study. Both glucose lowering therapies may have a certain effect on glucagon concentrations and, therefore, may also bias our resulting glucagon concentrations in T2D patients. However, we did not find statistically significant differences between the glucagon levels of patients which were using DPP IV inhibitors and GLP-1 receptor agonists and patients who did not use them (ELISA: *p* = 0.565; RIA: *p* = 0.331).

A limitation of this study is the small number of included participants. Further, it needs to be pointed out that the main aim of the study was to compare the ELISA and RIA measurement rather than comparing absolute glucagon levels in people with T1D, T2D or healthy individuals or the relative difference in glucagon levels between the three groups. To do the latter analysis, the cohorts would need to be matched for at least age, sex diabetes duration, comorbidities and treatment options potentially influencing glucagon levels would need to be taken into account. Strength of this study was the inclusion of three different groups, namely healthy individuals and T1D and T2D patients.

## 5. Conclusions

In conclusion, ELISA was able to observe a significant difference in glucagon levels between healthy individuals and T2D patients, which RIA did not. Additionally, in contrast to RIA, ELISA yielded lower glucagon levels in healthy individuals than in T1D and T2D patients. The overall median glucagon concentrations in all investigated groups were significantly higher when assessed with RIA than with the ELISA.

Our results revealed that the choice of a selective laboratory method for glucagon measurement is highly relevant for mechanistic endocrine research and the accurate interpretation of the resulting data.

## Figures and Tables

**Table 1:** Baseline characteristics.

	Healthy (*n* = 20)	Type 1 Diabetes (*n* = 20)	Type 2 Diabetes (*n* = 20)	*p*-Value
Sex (male/female)	7/13	15/5 ^#^	12/8	0.035
Age (years)	29.5 ± 6.5	35.5 ± 15.6	63.6 ± 8.9 *	0.000
Fasting plasma glucose (mg/dL)	87 ± 5	161 ± 59 ^#^	168 ± 57 ^#^	0.000
Diabetes duration (years)	-	18.2 ± 11.9	19.3 ± 10.2	
HbA1c (mmol/mol)	-	71 ± 17	64 ± 14	
Types of therapy (%)				
Insulin		100	80	
Metformin		0	55	
DPP IV inhibitor		0	35	
SGLT-2 inhibitor		0	10	
Sulfonylurea/Glinide		0	10/5	
GLP1-RA		0	10	

^#^*p* < 0.05 vs. Healthy; * *p* < 0.05 vs. Healthy and T1D.

## Data Availability

The dataset generated and analysed in this study is not publicly available but may be obtained from the corresponding author upon a reasonable report.
